# Scanning Electron Microscopic Evaluation of Smear Layer Removal Using Isolated or Interweaving EDTA with Sodium Hypochlorite

**DOI:** 10.22037/iej.2017.11

**Published:** 2017

**Authors:** Ângelo José da Silva Beraldo, Rogério Vieira Silva, Alberto Nogueira da Gama Antunes, Frank Ferreira Silveira, Eduardo Nunes

**Affiliations:** a *Department of Dentistry, Pontificial Catholic University of Minas Gerais, Belo Horizonte, Minas Gerais, Brazil*

**Keywords:** Root Canal Irrigants, Scanning Electron Microscopy, Smear Layer

## Abstract

**Introduction:**

The aim of this study was to verify the effect of alternating 2.5% sodium hypochlorite (NaOCl) and 17% ethylenediaminetetraacetic acid (EDTA) on the smear layer removal from root canal surfaces.

**Methods and Materials:**

A total of 15 single-rooted human teeth, instrumented with ProTaper files, were randomly distributed in 3 groups. In group 1 (*n*=7) the canals were irrigated with 1 mL of 2.5% NaOCl between files and final irrigation was done with 1 mL of 2,5% NaOCl, followe by 1 mL of 17% EDTA, for a perio of 15 sec with new irrigtion of 1 mL of 2,5% NaOCl at each change of files. In group 3 (control group) (*n*=1), saline solution was used. All samples were cleaved into two sections, metalized and analyzed under scanning electron microscopy (SEM). The presence or absence of smear layer in the cervical, middle and apical thirds, with scores varying from 1 to 3, respectively were evaluated. The data were submitted to nonparametric Mann-Whitney U test. The level of significance was set at 0.05.

**Results:**

It was observed that there was a greater discrepancy between groups with respect to the apical third. In the other areas there was a greater similarity between the scores attributed to the groups. There was a statistically significant difference between the groups only in the apical third, when group 1 presented the higher median (*P*<0.05).

**Conclusion:**

The alternating use of EDTA during instrumentation with NaOCl was the most effective irrigation method to remove the apical smear layer. Both forms of irrigation were effective on removal of the smear layer in the coronal and middle thirds of the canals.

## Introduction

The aim of endodontic therapy is to clean and eliminate the microorganisms from the root canal system. During instrumentation a mud like layer known as the smear layer (SL) is formed which contains organic and inorganic components from pulp, dentine, bacteria and their byproducts that occlude the dentinal tubules [1]. The smear layer has approximately 1-2 μm thickness and can also penetrate into the dentinal tubules to a depth of up to 40 μm [2]. Its presence may reduce dentinal permeability and attenuate or prevent the penetration of bacteria in the dentinal tubules [3]. Moreover, it can prevent or hinder the action of irrigating solutions and intracanal medication used between sessions, and even interfere with the penetration of filling materials within the dentinal tubules and their adaptation to the dentinal walls [4-6]. Thus, elimination of the smear is important after chemomechanical preparation of root canal system [5, 7-9].

Some methods, substances or techniques have been used to remove the smear layer [10-14]. Some authors recommended a combination of irrigating solutions that can change the organic and inorganic components of the smear layer [10-12]. Sodium hypochlorite (NaOCl) and ethylenediaminetetraacetic acid (EDTA) are effective when used together. EDTA chelates calcium, demineralizes dentin and removes the inorganic components of the smear layer, while NaOCl removes the organic material, including collagen matrix [15].

Different concentrations and variations in components of EDTA are reported in the literature [5, 16]. Although many authors indicate that EDTA should be used at the end of the instrumentation [17-20] the literature demonstrates variations in the volume of solution and irrigation time. The literature shows that these solutions must stay in contact with the canal walls from 30 sec to 10 min, and the longer the contact time, the more the progressive dissolution of the dentin with erosion potential in the peri- and intertubular area. [17, 21-24]. Thus, there is a need to verify different smear layer removal protocols in order to create a better approach when dealing with root canals preparation.

The aim of this *in vitro* scanning electron microscopy (SEM) study was to verify the efficacy of alternating use of 2.5% NaOCl and 17% EDTA in the removal of smear layer during the chemomechanical preparation of root canals.

## Materials and Methods

After the approval by the Ethics and Research Committee of the Pontifical Catholic University of Minas Gerais (PUC-MG), a total of 15 single-rooted permanent human teeth originating from the PUC-MG tooth bank were used. Following radiographic evaluation, the criteria for exclusion in the study were as follows: those who presented endodontic fillings, curved root canals, calcifications, nodules and more than one canal.

The teeth were stored in distilled water and 2.5% NaOCl in proportion of 10:1 and access cavities were prepared and the entrance of the root canals was located with endodontic probe (Odous, Belo Horizonte, Brazil).

To determine the working length (WL), a #10 K-file (Dentsply Maillefer, Ballaigues, Switzerland) was introduced in the canal until it was visible through the apical foramen. The WL was set 1 mm short of this length. For the canal instrumentation after negotiating the canals with size 10 and 15 K-files, S1 and S2 ProTaper rotary instruments were used followed by F1 and F2 finishing files in equal measures. 

During all preparation procedures, irrigation was performed with a 27 gauge cannula (Vista Dental, Racine, WI, USA) coupled to the 5 mL disposable syringe (Injex, Ourinhos, SP, Brazil). After using each file (manual or roundabout) the canals were irrigated with 1 mL of 2.5% NaOCl (Lenza Farmacêutica, Divisão Odontológica, Belo Horizonte, Brazil) and the specimens were divided into three groups:

In group 1 (*n*=7), samples were irrigated with 1 mL of 2.5% NaOCl at each change of files and irrigation with 1 mL of 17% EDTA (Lenza Farmacêutica, Divisão Odontológica, Belo Horizonte, Brazil) was also done; the latter solution was left in the canal for three min and was agitated with #10 K-file followed by final irrigation with 1 mL of 2.5% NaOCl.

In group 2 (*n*=7), irrigation was done with 1 mL of 2.5% NaOCl, followed by 1 mL of 17% EDTA, being stirred with #10 K-type file for a period of 15 sec with new irrigation of 1 mL of 2.5% NaOCl at each change of files. 

In group 3 (control group) (*n*=1), irrigation was done with saline (Lenza Farmacêutica, Divisão Odontológica, Belo Horizonte, Brazil).

All canals were dried with absorbent paper points (#F2 PaperPoint, Dentsply Maillefer, Ballaiguess, Switzerland). Then the teeth were grooved with a diamond disk (4217, DFS, Riedenburg, Germany) and split longitudinally using chisel and mallet. One half of each tooth was randomly chosen and placed using carbon tape in a circular metal stub measuring 10 mm in diameter and 5 mm in height. Then the samples were coated with gold for SEM evaluation (JEOL Co., Tokyo, Japan). 

One point was selected in each section to be evaluated at the canal cervical, middle and apical thirds under 1000× magnification. The presence or absence of smear layer was assessed by an experienced and calibrated examiner using the following scores: *score 1*; no smear layer with dentinal tubules unobstructed and clean, *score 2; *moderate smear layer, presence of debris observed only in the dentinal tubules, and *score 3*; accentuated, located in the root surface and in the dentinal tubules.

Data were tabulated and later submitted to nonparametric Mann-Whitney U test. The level of significance was set at 0.05. Tests were performed by the BioEstat 5.3 software (Instituto de Desenvolvimento Sustentável Mamirauá, Tefé, Brazil).

## Results

Table 1 shows the distribution of samples in the apical, middle and coronal thirds. It was observed that there was a greater discrepancy between groups with respect to the apical third (*i.e.* 71.42% of *score 3* in group 1, and 57.14% cases with *score 1* in group 2). In the other areas there was a greater similarity between the scores attributed to the groups. There was a statistically significant difference between the groups only in the apical third, when the group 1 presented the higher median (*P*<0.05). 

## Discussion

The advantages and disadvantages of smear layer removal are still controversial. The need and the importance of smear layer removal are connected to the root content (live or necrotic pulp) [21]. In case of treating vital teeth where there is no contamination and the aseptic chain is maintained, removal of the smear layer may not be required [20]. However, if treatment of a necrotic tooth is due, the smear layer will become infected, and the clinician should consider the importance of its removal [21].

In case of pulp necrosis, microorganisms penetrate into the dentinal tubules and can be found deeper within the dentin. In such circumstances, the smear layer covers the bacteria, making it difficult for the intra-canal medication to contact the walls of the canal or even to penetrate the dentinal tubules [3, 25]. Similarly, the smear layer will act as an intermediate physical barrier that can also interfere in the adhesion and penetration of filling material within the dentinal tubules. This situation could also happen in teeth with vital pulps [4, 5, 26]. Contradicting these assertions, Timpawat *et al*. [20], found that the removal of the smear layer caused a significant increase of the apical microleakage.

Aiming to further analyze the presence or absence of smear layer at the entrance and inside the dentinal tubules, after chemomechanical preparation, the SEM method has been the most widely used means for this purpose [5, 7, 16-18, 21, 26, 27]. However different magnifications have been used for this type of evaluation (500 and 700× by Goldman *et al.* [17]; 480, 960 and 1080× by Alaçam [28]; 200 and 400× by Peters and Barbakow [29]; 50 and 2000 by Crumpton *et al.* [30]. In the present study 1000× magnification was used, as like many other studies [19, 26, 31, 32]. The scoring system used in the present study is similar to other studies reported in the literature [19, 26, 28, 31]. At this magfication, the presence of smear layer can be detected clearly. In adition, no dessecation protocols were made because it is not known if they could remove the smear layer.

Various materials are recommended for the removal of the smear layer: EDTA [33 32]; MTAD [26]; EDTA-T [34]; 10% and 20% citric acid [35, 36]; Laser [37] and 2.5% or 5.25% NaOCl solution with ultrasonic [31].

The irrigating solutions used in this study were chosen due to their known properties such as removal of inorganic and organic content of smear layer. McComb and Smith [25] demonstrated that EDTA helps with smear layer removal. Goldman *et al.* [17] demonstrated that EDTA only removed the inorganic moiety when used alone; Yamada *et al.* [27], Garberoglio and Becce [32], Calt and Seper [24], Peres and Pourcel [16] and Teixeira *et al. *[19] independently showed that the alternate use of EDTA and NaOCl is an efficient method for removal of the organic and inorganic parts of smear layer.

The cleaning of the root canal is closely related to the penetration of irrigating cannulas inside the root canals. This procedure is used ideally as close as possible to the apical third, thus optimizing the action of irrigating (NaOCl and EDTA) throughout the working length [35]. Due to the small size of the root canal, air bubbles may frequently happen inside it while using chemical solutions, especially in the middle and apical thirds. This prevents its contact with the dentin walls, making it difficult to remove the smear layer. The frequent use of #10 K files throughout the working length, added to the more frequent use of EDTA for shorter periods. This may have been the major factor in obtaining better results from the use of EDTA and with NaOCl, especially in the apical third, where the removal of the smear layer has always been shown to be a hard procedure to perform [9, 38]. Furthermore, the contact of EDTA for shorter periods can lead to less deleterious effect on dentin, reducing the possibility of erosion [34]. Another interesting factor was that the total time of non-continuous presence of EDTA (3 min) was shorter than in the use of EDTA in a final irrigation [4]. The demineralizing effect of EDTA is proportional to its pH that is gradually modified and limits its demineralizing action. In high concentrations it can become irritating to periapical tissues. Prolonged use, besides participating in the removal of the smear layer, may demineralize peri- and intertubular dentin, leading to a reduction of its hardness. Another finding is that the dentin may become irregular, changing its surface tension and modifying the contact angle with the filling material, which could affect the quality of the filling [39].

**Table 1:** Absolute and relative frequency distribution of the smear layer scores

**Surface**	**Group (N=7)**	***Score 1 *** **N (%)**	***Score 2 *** **N (%)**	***Score 3*** ** N (%)**
**Apical third**	Group 1	1 (14.29)	1 (14.29)	5 (71.42)
	Group 2	4 (57.14)	3 (42.86)	0 (0.00)
**Middle third**	Group 1	1 (14.29)	6 (85.71)	0 (0.00)
	Group 2	5 (71.42)	1 (14.29)	1 (14.29)
**Coronal third**	Group 1	2 (28.57)	2 (28.57)	3 (42.86)
	Group 2	2 (28.57)	4 (57.14)	1 (14.29)

**Table 2.** Median, range and comparison of the smear layer scores between groups

**Experimental group**	**Surface**		
	**Apical third**	**Middle third**	**Coronal third**
**Group 1**	3 (1-3) a	2 (1-2) a	2 (1-3) a
**Group 2**	1 (1-2) b	1 (1-3) a	2 (1-3) a

a,b Within columns, medians followed by the same lowercase letter are not significantly different from each other (*P*>0.05). *P-values* were obtained by using the Mann-Whitney test (*P*<0.05)

The alternative use of NaOCl and EDTA showed better results in smear layer removal from the apical third of specimens in group 2. This observation seems of utmost importance because it is precisely in this region where the greatest difficulty in smear layer removal occurs. Both techniques were effective in the removal of smear layer in the middle and coronal thirds. The technique used in group 1 presented the same results of other studies [16, 18, 19].

The methodology used in group 2 opens an interesting perspective about the use of EDTA for a shorter period of time. In future studies, it would be significant to verify whether EDTA can be used in lower concentrations, which might minimize its deleterious effects in dentinal walls. Another factor to be investigated is to observe any interference of this association in the action of NaOCl, since cleaning, especially in the apical third, was relevant. Therefore, it opens a pathway for futures scientific researches.

## Conclusion

The alternating use of EDTA during instrumentation with NaOCl proved to be the most effective form of irrigation in the elimination of the smear layer from the apical third of the root canal system; both forms of irrigation have proven to be effective in the removal of the smear layer from the root canal system in the coronal and middle thirds of the samples.
